# Drug-related emergency department visits: external validation of an assessment tool in a general emergency department population

**DOI:** 10.1007/s11096-024-01760-8

**Published:** 2024-07-03

**Authors:** Lisbeth D. Nymoen, Julie L. S. Pettersen, Trude. E. Flatebø, Erik Øie, Kirsten K. Viktil

**Affiliations:** 1 Diakonhjemmet Hospital Pharmacy, Oslo, Norway; 2 Department of Pharmacy, University of Oslo, Oslo, Norway; 3 Department of Internal Medicine, Diakonhjemmet Hospital, Oslo, Norway

**Keywords:** Drug-related hospitalisation, Emergency departments, Medication review, Risk assessment tool, Validation of prediction model

## Abstract

**Background:**

The process of identifying drug-related hospitalisations is subjective and time-consuming. Assessment tool for identifying hospital admissions related to medications (AT-HARM10) was developed to simplify and objectify this process. AT-HARM10 has not previously been externally validated, thus the predictive precision of the tool is uncertain.

**Aim:**

To externally validate AT-HARM10 in adult patients admitted to the emergency department (ED).

**Method:**

This retrospective cross-sectional study investigated 402 patients admitted to the ED, Diakonhjemmet Hospital, Oslo, Norway. A trained 5th-year pharmacy student used AT-HARM10 to assess all patients and to classify their ED visits as possibly or unlikely drug-related. Assessment of the same patients by an interdisciplinary expert panel acted as the gold standard. The external validation was conducted by comparing AT-HARM10 classifications with the gold standard.

**Results:**

According to AT-HARM10 assessments, 169 (42%) patients had a possible drug-related ED visit. Calculated sensitivity and specificity values were 95% and 71%, respectively. Further, positive and negative predictive values were 46% and 98%, respectively. Adverse effects/over-treatment and suboptimal treatment were the issues most frequently overestimated by AT-HARM10 compared with the gold standard.

**Conclusion:**

AT-HARM10 identifies drug-related ED visits with high sensitivity. However, the low positive predictive value indicates that further review of ED visits classified as *possible* drug-related by AT-HARM10 is necessary. AT-HARM10 can serve as a useful first-step screening that efficiently identifies unlikely drug-related ED visits, thus only a smaller proportion of the patients need to be reviewed by an interdisciplinary expert panel.

## Impact statements

- The findings from this study indicate that AT-HARM10 can be useful as a first-step screening; unlikely drug-related ED visits are ruled out directly from further review, leaving a smaller proportion of the patients who need to be reviewed by for instance an interdisciplinary expert panel.
- In patients where AT-HARM10 identifies possible drug-related ED visits related to adverse effects/over-treatment and suboptimal treatment, the tool often overestimates the drug contribution.
- To avoid a large number of false positives, an interdisciplinary expert panel should discuss patients classified as possible drug-related ED visits by AT-HARM10 to conclude on the true prevalence

## Introduction

It is estimated that up to 70% of drug-related emergency department (ED) visits may be preventable [1–3]. Drug-related ED visits and hospital admissions have negative clinical consequences for the patients and economic consequences for the healthcare system and society [4–6]. Thus, reducing, avoiding, or preventing drug-related ED visits and hospital admissions is a preferred outcome for research investigating the impact of medication review interventions [7]. Paradoxically, a universal definition of drug-related ED visits and hospital admissions does not exist. In addition, the process of identifying these events is often subjective, time-consuming, and varies between studies [8, 9]. With the aim of simplifying and objectifying the identification of drug-related ED visits/hospital admissions, different risk assessment tools have been developed during the last decades [8, 10, 11].

*Assessment tool for identifying hospital admissions related to medications (AT-HARM10)* is one of the recently developed risk assessment tools [8]. AT-HARM10comprises ten yes or no questions, which should be answered to determine whether the admission is *unlikely* or *possible* drug-related [8]. AT-HARM10 is less time-consuming compared with the *Drug-related hospital admissions (DRA) Adjudication Guide* [10], with mean reported time spent on assessment per patient of 5.7 min and 23 min, respectively [8, 10]. A recent study revealed that AT-HARM10 and DRA Adjudication Guide agree to a high degree (95%) on what patients have a possible drug-related hospital admission when applied to the same population [12]. Assessments with AT-HARM10 does not require implementation of computer software as the Quick assessment of drug-related admissions over time (QUADRAT) tool [11]. Unlike AT-HARM10, the 307 trigger flags of the QUADRAT tool cannot be applied and evaluated manually. Hence, to use the QUADRAT tool, implementation of triggers/flags in the electronic patient journal or national databases is required [11].

AT-HARM10 was developed to identify drug-related hospital admissions in older patients (aged 65 years or older) [8]. AT-HARM10 has also been validated to identify drug-related readmissions [13] and tested for applicability and reliability regarding drug-related ED visits in older patients (aged 65 years or older) [14]. AT-HARM10 has, however, not previously been externally validated or validated in an ED population also including younger adult patients (aged 18 years or older).

### Aim

The aim of this study was to externally validate AT-HARM10 in adult patients admitted to the ED.

### Ethics approval

Patients included in the present study were included in a prior randomized controlled trial (RCT) [15]. These patients were also included in a prior sub-study used as the gold standard in the present study [9]. Both the prior RCT and the sub-study were approved by the institutional review board and the Norwegian Regional Committee for Medical and Health Research Ethics (Case number: 2015/1356/REK Helse south-east A, 27.08.2015) [15]. Written informed consent was obtained from all patients before inclusion [15]. No further ethical approval was necessary to conduct the present study. To ensure transparency, the present study was designed and reported according to thetransparent reporting of a multivariable prediction model for individual prognosis or diagnosis (TRIPOD) statement [16].

## Method

### Study design and setting

**Fig. 1:**
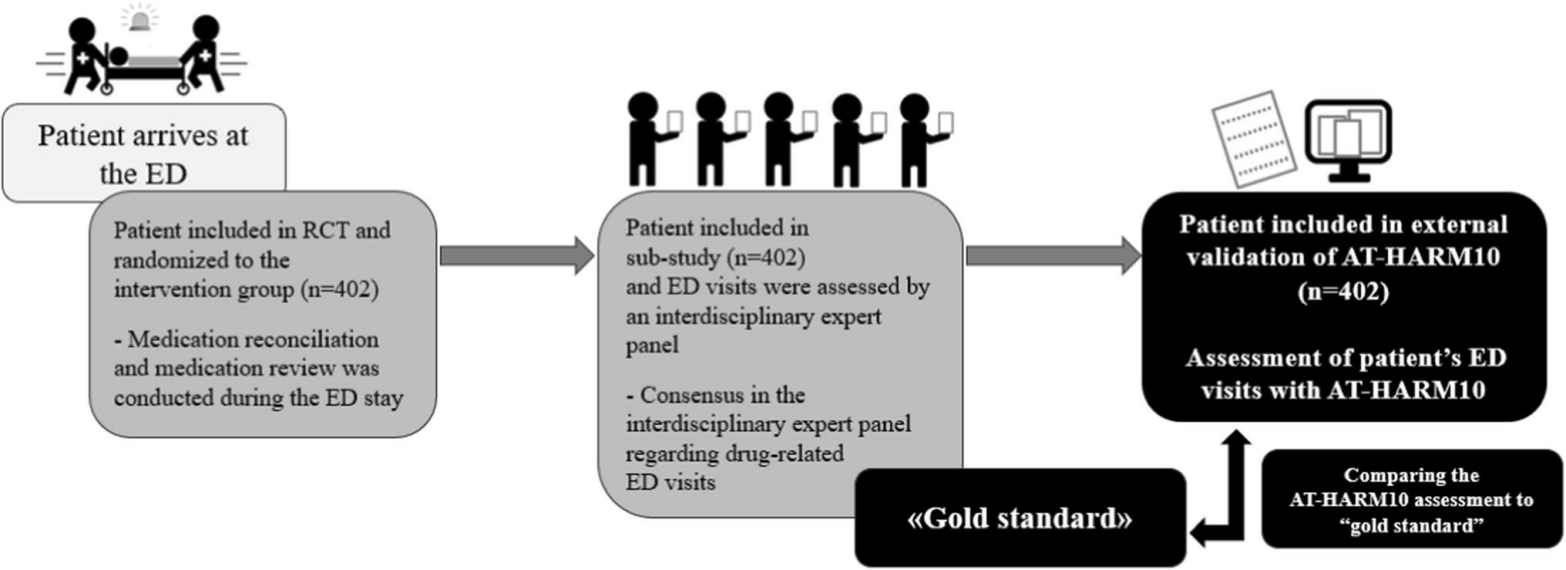
Patient inclusion to external validation of Assessment tool for identifying hospital admissions related to medications (AT-HARM10). Patients were included to a randomized controlled trial (RCT) [15] at arrival to the emergency department (ED). Patients randomized to the intervention group were further included in a sub-study [9]. The intervention group patients were finally included to the external validation of AT-HARM10 and results from the sub-study was used as “gold standard”

Through this retrospective cross-sectional study, an external validation of AT-HARM10 was conducted [8]. Patients admitted to the ED at Diakonhjemmet Hospital, from April 2017 to May 2018, included and randomized to the intervention group (*n* = 402) in a prior RCT were included [15]. Results from a previous sub-study of the intervention group patients from the RCT was set as the gold standard in the presented external validation of AT-HARM10 [9]. AT-HARM10 assessments were conducted from September 2022 to January 2023. Figure 1 illustrates patient inclusion to the presented study and the gold standard is explained thoroughly in a separate section.

In Norway, general practitioners (GPs) and the municipal emergency clinics have a gatekeeper function and handle patients with non-severe conditions. More severe conditions are referred to a hospital’s ED. The referring healthcare personnel set a tentative referral reason after assessing the patient’s symptoms and conducting an initial examination (before the patient arrives at the ED). Diakonhjemmet Hospital is a local, urban hospital in Oslo, Norway. Patients 18 years or older with both medical and surgical symptoms are referred to the ED at Diakonhjemmet Hospital. In the previous RCT, all patients arriving at the ED, willing to/capable of providing written, informed consent were eligible for inclusion. The intervention of the RCT, comprising a medication reconciliation and a medication review was carried out during the patients’ ED visits [15].

### Assessment with AT-HARM10

The assessments in the present study were conducted by a 5th-year pharmacy master student. The student received training on how to use AT-HARM10 from two experienced clinical pharmacists before study start.

As only one student conducted AT-HARM10 assessments in the present study, some actions were taken to verify the results; (1) The first 20 included patients were assessed by the student and an experienced clinical pharmacist individually. Their AT-HARM10 classifications were compared retrospectively to validate AT-HARM10 classifications. The predefined limit of agreement ≥ 80% was achieved. (2) The student continued assessing all patients according to AT-HARM10 methodology [8], and the experienced clinical pharmacist assessed every 4th patient. AT-HARM10 classifications were compared retrospectively for verification, and the calculated inter-rater reliability (IRR) was found to be substantial throughout the whole data material (Cohen’s kappa 0.74) [17].

Throughout the assessments, the student registered AT-HARM10 classification (unlikely or possible drug-related) for each patient’s ED visit. If the ED visit was classified as possible drug-related, the student registered what drug(s) were suspected to be involved.

According to Kempen et al. AT-HARM10 assessment is finalised as soon as any of the ten questions are answered with “yes” [8]. To get a better understanding of the strengths and limitations of AT-HARM10, the student answered and reported all ten AT-HARM10 questions regardless of how many questions were answered with “yes”. This approach gave a more nuanced impression of how the tool classifies ED visits and enabled further review of what questions were most frequently answered with “yes” (i.e. issues where AT-HARM10 is sensitive).

Planned ED visits were not possible to classify with AT-HARM10 [8], due to the wording in the questions. Thus, the planned ED visits were marked as “planned” by the student during the assessment.

### Available information during AT-HARM10 assessment

Information about each included patient and their ED visit was presented in deidentified patient forms (Template in Online Resource 1 of a prior publication [9]). The forms comprised demographic data (age, sex, who administered the patient’s drugs, and a brief prior medical history), data regarding the ED visit (triage status, referral reason, medical or surgical referral reason, laboratory results from the ED), a reconciled drug list, drug-related problems identified through medication review conducted in the ED and final diagnosis documented in the discharge summary. The forms did not disclose whether the patients were admitted to a hospital ward after their ED visit or not. These forms were available to the student during AT-HARM10 assessments.

### Gold standard

In the previous sub-study an interdisciplinary expert panel, consisting of two chief physicians and three experienced clinical pharmacists, assessed the 402 patients to classify their ED visit as drug-related or non-drug-related [9]. The expert panel first assessed all ED visits individually before meeting to discuss each ED visit and trying to reach consensus. The interdisciplinary expert panel used the same patients forms as the student in the present study as information basis when assessing the ED visits [9]. Due to lack of information or disagreement within the interdisciplinary expert panel, 10 (2.5%) patients could not be classified [9]. Hence, consensus was reached in 392 patients, 79 (19.7%) of the included patients had a drug-related ED visit and 313 patients had a non-drug-related ED visit [9]. This consensus (*n* = 392) was set as the gold standard in the presented external validation of AT-HARM10.

The student conducting AT-HARM10 assessments was blinded to the expert panel classification whilst conducting the assessments [9].

### Statistics

Data handling was conducted in Microsoft Office Excel 365. Statistical analyses were carried out in Stata SE version 16. Demographic statistics are given as n and percentage for categorical variables, and median, interquartile range (IQR), and range for continuous variables, due to the skewed distribution of these variables.

Drugs listed in the patients’ reconciled drug lists were classified according to the Anatomical Therapeutic Chemical (ATC)-classification [18], and analysed on ATC 3rd level (ATC3-groups). The relative frequency of ATC3-groups was calculated as follows: how often a drug from the specified ATC3-group was involved in a possible drug-related ED visit divided by the number of times drugs from that specific ATC3-group were used in the study population.

Sensitivity, specificity, positive predictive value, and negative predictive value of AT-HARM10 were calculated by comparing AT-HARM10 classifications with the gold standard, and according to standard equations. Validation of AT-HARM10 comprised patients who had been classified during assessment with AT-HARM10 and where the interdisciplinary expert panel reached consensus (*n* = 379).

$$ Sensitivity = \frac{True\,positive}{{True\,positive + False\,negative }} \times 100\quad Specificity = \frac{True\,negative}{{False\,positive + True\,negative }} \times 100 $$

$$ Positive\,predictive\,value = \frac{True\,positive}{{True\,positive + False\,positive }} \times 100\quad Negative\,predictive\,value = \frac{True\,negative}{{False\,negative + True\,negative }} \times 100 $$

## Results

Demographics of the 402 included patients are presented in Table 1. A total of 169 (42.0%) patients had a possible drug-related ED visit according to AT-HARM10 assessment (Table 2). Thirteen (3.2%) patients could not be classified with AT-HARM10 as their ED visits were planned (Table 2).

**Table 1:** Demographics of study population (*n* = 402)

Age	Median (IQR, range)	67 (27, 19–96)
	Patients ≥ 65 years *n* (%)	220 (54.7)
Sex	Female *n* (%)	192 (47.8)
	Male *n* (%)	210 (52.2)
Referral reason allocation	Medical *n* (%)	280 (69.7)
	Surgical *n* (%)	122 (30.4)
Patients admitted to DH last 12 months before ED visit *n (*%)	229 (57.0)	
Number of prescribed drugs†	Regular drugs, median (IQR, range)	4 (6, 0–19)
	Patients using ≥ 5 regular drugs n (%)	178 (44.3)
	As needed drugs, median (IQR, range)	2 (3, 0–9)
Responsible for drug administration before ED visit	Patient *n* (%)	335 (83.3)
	Other (next in kin/home care service/nursing home) *n* (%)	67 (16.7)
Hospitalised patients†† *n* (%)		273 (67.9)

*DH* Diakonhjemmet Hospital, *ED* emergency department

^†^Number of prescribed drugs obtained through medication reconciliation

^††^The other part was discharged directly from the ED

**Table 2:** Classification of emergency department (ED) visits with AT-HARM10

Classification	Number of patients (%) *n* = 402
Possible drug-related ED visits	169 (42.0)
Unlikely drug-related ED visits	220 (54.7)
Not classified patients (planned visits)	13 (3.2)

A broad spectrum of drugs was involved in the possible drug-related ED visits identified with AT-HARM10. Agents acting on the renin-angiotensin system (RAS-inhibitors) were the ATC3-group most frequently involved in possible drug-related ED visits (22.5%) (Table 3). Further, beta-blocking agents and antithrombotic agents were each involved in 17.2% and 16.0% of the possible drug-related ED visits, respectively. Antineoplastic agents, immunosuppressants, and hormone antagonists were the ATC3-groups with the highest relative frequency of possible drug-related ED visits (Table 3).

**Table 3:** ATC3-groups involved in drug-related ED visits

ATC3-group		Relative frequency of drug-related ED visits in ATC3-groups† %	Proportion of drug-related ED visits caused by specific ATC3-groups (*n* = 169) *n* (%)
Antineoplastic agents	L01A/C/X	87.5	7 (4.1)
Immunosuppressants	L04A	70.6	12 (7.1)
Hormone antagonists and related agents	L02B	60.0	3 (1.8)
Corticosteroids for systemic use, plain	H02A	44.2	19 (11.2)
Antiarrhythmics, class I and III	C01B	42.9	3 (1.8)
Agents acting on the renin-angiotensin system, with or without thiazide (RAAS-inhibitor)	C09A/B/C/D	36.5	38 (22.5)
Diuretics	C03C/E	34.7	17 (10.1)
Beta-blocking agents	C07A	29.0	29 (17.2)
Inhalants for obstructive airway diseases, both adrenergic and others	R03A/B	20.4	23 (13.6)
Antithrombotic agents	B01A	14.2	27 (16.0)

Included ATC3-groups in the table: either contributed to 15 or more possible drug-related ED visits, have a relative frequency > 35%, or both

A single drug-related ED visit could involve drugs from multiple ATC3-group and also multiple drugs from the same ATC3-group. ATC3-codes of the presented ATC3-group can be found at www.whocc.no/atc_ddd_index/

*ATC* Anatomical therapeutic chemical-classification of drugs

^†^The relative frequency was calculated as follows: how often a drug from the specified ATC3-group was involved in possible drug-related ED visits divided by number of times drugs from that specific ATC3-group were used by the 389 classified patients

### Validation of AT-HARM10 with “gold standard”

Calculated sensitivity and specificity values for AT-HARM10 compared with gold standard were 95% and 71%, and positive and negative predictive values were 46% and 98%, respectively (Table 4).

**Table 4:** External validation of assessment tool for identifying hospital admissions related to medications (AT-HARM10)

AT-HARM10 assessment	Consensus interdisciplinary expert panel (gold standard)			
	Drug-related ED visit	Non-drug-related ED visit	Unresolved/no consensus	Total
Possible drug-related visit	74 (true positive)	87 (false positive)	8	169
Unlikely drug-related visit	4 (false negative)	214 (true negative)	2	220
Planned visit (not classified)	1	12	0	13
Total	79	313	10	402

Calculations of sensitivity, specificity, positive predictive value, and negative predictive value included patients who had been classified during assessment with AT-HARM and the interdisciplinary expert panel reached consensus (gold standard) (*n* = 379), presented as numbers in highlighted cells

*ED* emergency department

It was investigated what questions were most frequently answered with “yes” among the 87 patients classified with a possible drug-related ED visit in AT-HARM10 assessment and non-drug-related by the interdisciplinary expert panel (false positive, Table 4). The questions related to adverse effects/over-treatment and suboptimal treatment was answered “yes” for 65 (75%) and 39 (45%) of the patients, respectively.

## Discussion

### Statement of key findings

In this external validation of AT-HARM10, it was revealed that 42.0% of the assessed ED visits were classified as possible drug-related with AT-HARM10. When comparing AT-HARM10 classification with the gold standard the revealed sensitivity, specificity, and negative predictive values for the tool were high; however, the positive predictive value was low.

### Strengths and weaknesses

Given the single study location in one specific healthcare system, where patients are referred to the hospital’s ED, the results are not necessarily generalisable to EDs in other countries. The prevalence of drug-related ED visits revealed through AT-HARM10 assessments is, however, in line with previous studies [8, 14].

AT-HARM10 assessments in this study were performed in a large patient sample (*n* = 402), which strengthens the validity of the result.

Only one student assessed all ED visits in this study which may have caused bias. However, more than 25% of the patients was also assessed by an experienced clinical pharmacist, and the inter-rater agreement was substantial.

In this study, the information about the patients and their ED visits available to the student during AT-HARM10 assessments was limited to the patient forms, this may have influenced the assessments. However, the same patient forms were also used by the interdisciplinary expert panel in the previous study which constituted the gold standard [9]. The patient forms included information gained through medication reconciliation and medication review which increased the quality of the available information.

### Interpretation

#### Validation of AT-HARM10

During the development of the tool, Kempen et al. conducted an internal validation of AT-HARM10 and reported sensitivity, specificity, and negative predictive values which were in line with the values presented in the present study [8]. However, the reported positive predictive value in Kempen et al.’s study was higher [8].

The major reason for difference in positive predictive value between the present study and Kempen et al.’s study is the registered prevalence of drug-related admissions/ED visits identified by the gold standard [8]. The gold standard used in the present study identified a prevalence of drug-related ED visits at 19.7% versus 50% drug-related hospital admissions in Kempen et al.'s study [8, 9]. The higher prevalence revealed by Kempen et al. can be explained by higher age amongst the included patients, as age is a known risk factor for drug-related ED visits/hospital admissions [2, 19–22]. Kempen et al. only included patients older than 65 years old [8], whereas in the present study, only approximately 50% of the patients were 65 years or older.

The definition of a drug-related ED visit/hospital admission is similar in Kempen et al.’s study and the gold standard of the present study. The expert panels of both studies used an implicit assessment to identify drug-related ED visits/hospital admissions. However, Kempen et al. used an expert panel consisting of one physician and one clinical pharmacist [8], whereas the expert panel providing the gold standard in the present study consisted of two physicians and three clinical pharmacists [9], which may have influenced the prevalence.

Patients with a drug-related ED visit are more frequently admitted to hospital following their ED stay compared with patients with a non-drug-related ED visit [2, 9, 23]. In general, the prevalence of drug-related hospital admissions therefore, most likely is higher than the prevalence of drug-related ED visits. Which may be another important reason for the higher prevalence revealed by Kempen et al.’s expert panel compared with the gold standard of the present study.

#### Content validity

The prevalence of drug-related ED visits in earlier studies varies between 2.3 and 28.6% [2, 3, 9, 19, 23–25]. Hence, the prevalence revealed with AT-HARM10 in the present study is substantially higher in comparison. The two previous studies assessing admissions with AT-HARM10 found the prevalence of possible drug-related admissions to be 28.8% in the ED and 50% in hospital ward [8, 14].

In the present study, it was revealed that differences between classification with AT-HARM10 and the gold standard were mainly associated with AT-HARM10 identifying issues related to adverse effects/over-treatment and suboptimal treatment. This may indicate that AT-HARM10 is too sensitive regarding these drug-related problems or that adverse effects/ overtreatment and suboptimal treatment may be too complex issues to precisely classify objectively. Coppes et al. revealed that students using assessment tools often concluded on drug-related readmissions in patients where an expert panel found disease progression more profound than the contribution of medication [13]. A discussion within an interdisciplinary expert panel may result in a more realistic estimation regarding drug-related ED visits/hospital admissions based on experience and different knowledge gathered together. In an assessment tool such as AT-HARM10, it is for instance difficult to completely rule out adverse drug reactions in patients admitted with symptoms of heart failure who use beta-blocking agents, even though disease progression is more likely. Comparing what drug groups were most frequently involved in drug-related ED visits in the “gold standard” study and the present study, it is confirmed that for instance beta blocking agents are more frequently involved when using AT-HARM10 in assessments compared with the expert panel assessment [9].

#### Strengths and limitations of AT-HARM10

Assessments conducted with AT-HARM10 are less time- and resource-consuming compared with assessments conducted by an interdisciplinary expert panel [8, 9]. Also compared with the use of other assessment tools, AT-HARM10 is less time-consuming [8, 10].

The fact that AT-HARM10 classifies a large number of false positive drug-related ED visits compared with the interdisciplinary expert panel consensus is concerning. The other assessment tools, DRA Adjudication Guide and QUADRAT, have either been found to identify approximately the same patients as AT-HARM10 or have reported a positive predictive value in line with the value in the present study [11, 12]; hence, there is no evidence that these are superior to AT-HARM10.

It was revealed that AT-HARM10 identifies a high percentage of true positives and efficiently rules out true negatives. Hence, it is expedient to use AT-HARM10 as a first screening to rule out the *unlikely* drug-related ED visits directly. However, due to the high percentage of false positives, it is necessary to review the *possible* drug-related ED visits further for instance through an interdisciplinary expert panel discussion. The advantage of using AT-HARM10 as a first screening is that the expert panel only needs to review a smaller proportion of the patients, which could increase the feasibility of assessing drug-related ED visits in future studies.

### Further research

This external validation of AT-HARM10 revealed that drug-related ED visits are identified with high sensitivity. Hence, future studies aiming to determine the impact of medication review interventions can use AT-HARM10 as a first step towards revealing the prevalence of drug-related ED visits, admissions or readmissions. However, according to the results of this study, it is recommended that an interdisciplinary expert panel discuss the patients classified with as possible drug-related ED visit/admission to determine the true prevalence.

In terms of deciding what risk assessment tool most precisely estimates the prevalence of drug-related admissions, both the QUADRAT tool and the DRA Adjudication Guide still need external validation.

## Conclusion

In this first external validation of AT-HARM10, it was revealed that AT-HARM10 had an acceptable sensitivity, specificity, and negative predictive value. However, the revealed positive predictive value was low. Assessments conducted with AT-HARM10 are more time efficient compared with assessments conducted with other assessment tools and by interdisciplinary expert panels. AT-HARM10 is also intuitive in use and validated for use by students. Hence, the tool can be used as screening to remove unlikely drug-related ED visits directly, and thus, only a smaller proportion of the patients (*possible* drug-related ED visits) needs to be reviewed by an interdisciplinary expert panel.
